# Roles of the Two-MnSOD System of *Stenotrophomonas maltophilia* in the Alleviation of Superoxide Stress

**DOI:** 10.3390/ijms20071770

**Published:** 2019-04-10

**Authors:** Herng-Woei Jair, Hsu-Feng Lu, Yi-Wei Huang, Sz-Yun Pan, I-Ling Lin, Hsin-Hui Huang, Tsuey-Ching Yang

**Affiliations:** 1 Department of Clinical Pathology, Cheng Hsin General Hospital, Taipei 11220; Taiwan; jcm5851@gmail.com (H.-W.J.); ch1835@chgh.org.tw (H.-F.L.); 2 Department of Restaurant, Hotel and Institutional Management, 24205, Fu-Jen Catholic University, New Taipei City 24205, Taiwan; 3 Department of Biotechnology and Laboratory Science in Medicine, 11221, National Yang-Ming University, Taipei 11221, Taiwan; r19817037@yahoo.com.tw (Y.-W.H.); sarapanpan0208@gmail.com (S.-Y.P.); e8201212002@gmail.com (I.-L.L.); toe3273917@outlook.com (H.-H.H.)

**Keywords:** superoxide dismutase, MnSOD, FeSOD, SodA, *Stenotrophomonas maltophilia*, oxidative stress, SoxR

## Abstract

Manganese-dependent superoxide dismutase (MnSOD, SodA) and iron-dependent SOD (FeSOD, SodB) are critical cytosolic enzymes for alleviating superoxide stress. Distinct from the singular *sodA* gene in most bacteria, *Stenotrophomonas maltophilia* harbors two *sodA* genes, *sodA1* and *sodA2*. The roles of SodA1, SodA2, and SodB of *S. maltophilia* in alleviating superoxide stress were investigated. The expression of *sod* genes was determined by promoter–*xylE* transcriptional fusion assay and qRT–PCR. *SodA2* and *sodB* expressions were proportional to the bacterial logarithmic growth, but unaffected by menadione (MD), iron, or manganese challenges. SodA1 was intrinsically unexpressed and inducibly expressed by MD. Complementary expression of *sodA1* was observed when *sodA2* was inactivated. The individual or combined *sod* deletion mutants were constructed using the gene replacement strategy. The functions of SODs were assessed by evaluating cell viabilities of different *sod* mutants in MD, low iron-stressed, and/or low manganese-stressed conditions. Inactivation of SodA1 or SodA2 alone did not affect bacterial viability; however, simultaneously inactivating *sodA1* and *sodA2* significantly compromised bacterial viability in both aerobic growth and stressed conditions. SodA1 can either rescue or support SodA2 when SodA2 is defective or insufficiently potent. The presence of two MnSODs gives *S. maltophilia* an advantage against superoxide stress.

## 1. Introduction

Aerobic metabolism is the major method by which aerobic bacteria acquire energy for growth. Superoxide is an intracellular by-product of aerobic metabolism. In some instances, the intracellular superoxide level is further increased by exogenous stresses, which may subvert the electron transfer chains of bacteria and enhance the electron flow towards oxygen. The exogenous stress can arise from the environment or host cells [1]. Superoxides are detrimental to bacteria as they can inactivate some metalloenzymes by oxidizing their metal cofactor and in turn lead to bio-molecule dysfunction [2]. Therefore, aerobic microbial pathogens have developed an array of specialized enzymatic defense systems to protect them from superoxide attack [3]. Superoxide dismutases (SODs) catalyze the conversion of superoxide to hydrogen peroxide and oxygen, and this conversion is the first-line defense mechanism to protect bacteria from the harmful effects of superoxide [4]. 

SODs are metalloenzymes. Based on the metal cofactor present, SODs are classified into several families, with the most common amongst bacteria being MnSOD (using manganese as cofactor), FeSOD (using iron as cofactor), and Cu-ZnSOD (using copper and zinc ions as cofactors), which are encoded by three distinct genes termed as *sodA*, *sodB*, and *sodC*, respectively [5]. With few exceptions, SodA and SodB are restricted to intracellular/cytoplasmic compartments, while Cu-containing SodC is exclusively in the extracellular/periplasmic space. Importantly, since superoxide cannot freely diffuse across membranes, each compartment requires its own unique SOD enzyme to deal with superoxide stress in gram-negative microbial pathogens. The extracellular SODs (SodCs) are particularly important for eliminating superoxide produced by host cells, whereas the intracellular SODs (SodAs and SodBs) are thought to act mostly on superoxides generated as byproducts of aerobic metabolism. However, the intracellular superoxide level can be further increased by some stresses [1]. Therefore, the intracellular SODs should have a supplementary potency to deal with the exacerbated superoxide stress that accumulates in addition to aerobic metabolism-derived superoxide.

The classes and number of SODs in different microorganisms vary. For example, there are three SODs in *Escherichia coli* (SodA, SodB, and SodC) [6], two in *Pseudomonas aeruginosa* (SodA and SodB) [7], two in *Staphylococcus aureus* (SodA and SodM) [8], and one in *Streptococcus thermophiles* (SodA) [9]. These SODs differ in their metal requirements, location, and temporal expression. For bacteria harboring multiple SODs, SodA generally accounts for a large portion of the superoxide dismutase activity. Aside from some gram-positive bacteria such as *Bacillus cereus*, *Bacillus anthracis*, and *S. aureus*, SodA is a single-copy gene in most bacteria [8,10,11].

*Stenotrophomonas maltophilia*, an aerobic, gram-negative bacterium, is frequently isolated from soil, water, animals, the rhizosphere, and hospital equipment [12]. It is also commonly isolated from the respiratory tract of patients with cystic fibrosis (CF). CF is caused by a mutation in the cystic fibrosis transmembrane conductance regulator (CFTR) gene resulting in an increased mucous viscosity in CF lungs. CF epithelial airway cells consume a greater amount of O_2_ than normal cells, generating a microenvironment of intense oxidative stress. In view of its ubiquitous distribution, *S. maltophilia* must be able to adapt rapidly to a variety of environmental stresses. Based on comprehensive genome comparisons, *S. maltophilia* appears to carry a more complex SOD system compared with those of *E. coli* and *P. aeruginosa*. Distinct from the three SODs (SodA, SodB, and SodC) in *E. coli* and the two SODs (SodA and SodB) in *P. aeruginosa*, the five putative *sod* genes found in the *S. maltophilia* genome include two *sodA* genes (*sodA1* and *sodA2*), a *sodB* gene, and two *sodC* genes [13]. A noteworthy feature of *S. maltophilia* is that it possesses two genes encoding MnSODs, which differs from the general trend that gram-negative bacteria harbor only a single copy of the *sodA* gene. However, the roles of the two *sodA* genes in *S. maltophilia* are unclear. In this study, we aimed to understand the differential roles of the three cytosolic SODs in *S. maltophilia* with an emphasis on the two MnSODs.

## 2. Results

### 2.1. Bioinformatics Analysis of Putative SODs in S. maltophilia

**Figure 1:**
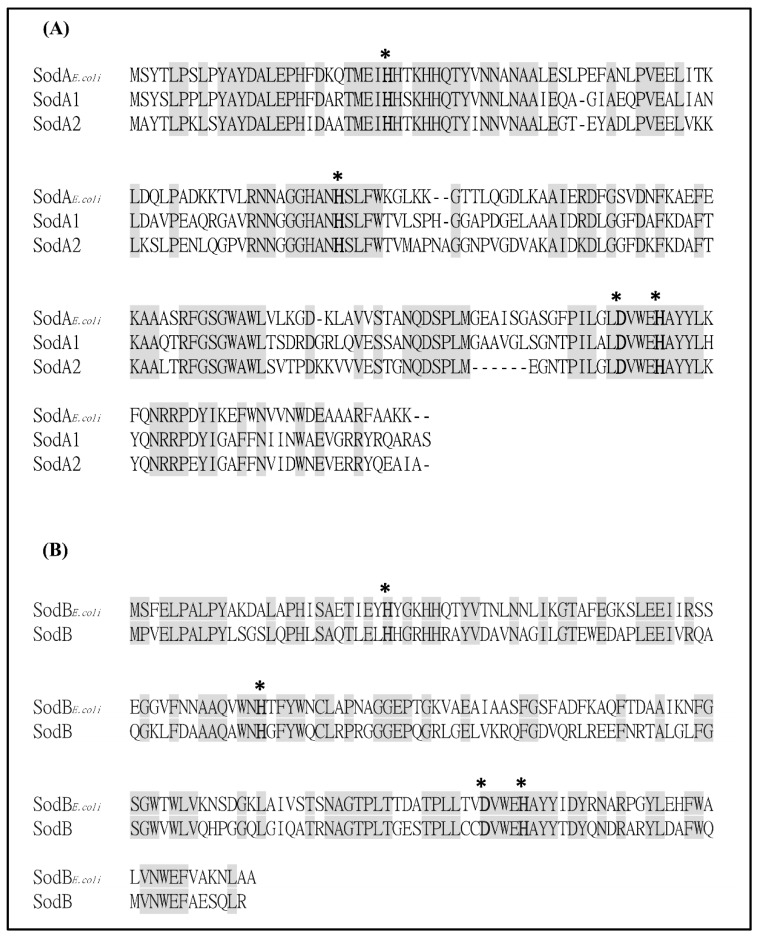
Proteins alignment of manganese-dependent superoxide dismutases (MnSODs) and iron-dependent SODs (FeSODs) of *E. coli* and *S. maltophilia*. Consensus amino acid residues are marked in gray. Residues marked with an asterisk are ligands to the metal cofactor. (**A**) Protein alignment of MnSODs of *E. coli* and *S. maltophilia*. (**B**) Protein alignment of FeSODs of *E. coli* and *S. maltophilia*.

Whole-genome sequencing of *S. maltophilia* K279a revealed the presence of five putative *sod* genes that encode two MnSODs (Smlt2828 and Smlt3238), one FeSOD (Smlt1616b), and two Cu-ZnSODs (Smlt0160 and Smlt0161) [13]. Of these, the MnSODs and FeSOD are cytosolic enzymes. As bacteria possessing two MnSODs are less common in gram-negative bacteria, and isoenzymes in bacteria may have overlapping functions, we were interested in determining the roles of Smlt2828 (named as *sodA1*) and Smlt3238 (named as *sodA2*) in oxidative stress response. SodA1 and SodA2 share 63% and 61% protein identities, respectively, to *E. coli* SodA and both share 68% protein identity. The critical residues which are ligands to metal binding are conserved in SodA1, SodA2, and SodB (Figure 1). Considering the possible functional redundancy between MnSOD and FeSOD, three SODs, SodA1, SodA2, and SodB, were simultaneously characterized in this study.

### 2.2. Implication of MnSODs and FeSOD in Aerobic Growth Conditions

**Figure 2:**
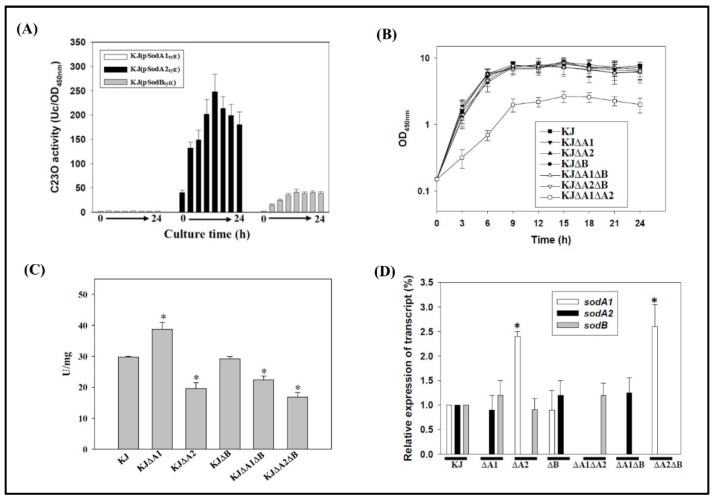
Implications of the three SODs in aerobic metabolism condition. (**A**) *Sod* genes expression of aerobically grown *S. maltophilia*. Overnight cultured KJ(pSodA1_xylE_), KJ(pSodA2_xylE_), and KJ(pSodB_xylE_) were inoculated into fresh Luria–Bertani (LB) medium at an initial OD_450nm_ of 0.15. Catechol 2, 3-dioxygenase (C23O) activities were monitored in 3-h time intervals for 24 h. Bars represent the mean from three independent experiments. Error bars represent the standard error (SD) of the mean. Data are expressed as mean ± SD (*N* = 3). (**B**) Bacterial growth of wild-type KJ and its derived *sod* mutants in LB broth. The bacterial growth was monitored by recording the OD_450nm_ every 3 h. Data are expressed as mean ± SD (*N* = 3). (**C**) SOD activities of wild-type KJ and its derived *sod* mutants. The mid-log phase bacterial cellular lysates were used for SOD activity assays. Bars represent the mean from three independent experiments. Error bars represent the SD of the mean. Data are expressed as mean ± SD (*N* = 3). * *p* < 0.001, significance calculated by the Student’s *t*-test. (**D**) Compensatory expression of other *sod* genes in *sod* mutants. Overnight cultured strains were inoculated into fresh LB medium at an initial OD_450nm_ of 0.15. After the 5-h culture, the *sodA1*, *sodA2*, and *sodB* transcripts were quantified by qRT–PCR. Bars represent the mean from three independent experiments. Error bars represent the SD of the mean. Data are expressed as mean ± SD (*N* = 3). * *p* < 0.001, significance calculated by the Student’s *t*-test.

The expression of some SODs is related to bacterial growth phase. To determine whether *sodA1*, *sodA2*, and *sodB* gene expression is associated with bacterial growth, expression of *sodA1*, *sodA2*, and *sodB* was individually analyzed using the promoter–*xylE* transcriptional fusion plasmids, pSodA1_xylE_, pSodA2_xylE_, and pSodB_xylE_. The catechol 2, 3-dioxygenase (C23O) activities expressed by these plasmids in wild-type KJ were monitored in a 24-h growth cycle. The results indicated that *sodA2* and *sodB* expression increased in a growth-dependent manner, with the highest expression occurring during the late logarithmic phase and *sodA2* expression being the most apparent. In contrast, the expression of *sodA1* was minimal during the 24-h monitored time course (Figure 2A).

For functional surveys of the three SODs, we constructed mutants in which the three SOD genes were inactivated either individually or in combination. Since the transcriptional organization of these SOD gene loci had not yet been characterized, in-frame deletion mutants were constructed to create stable nonpolar mutations. The *sodA1*, *sodA2*, and *sodB* triple mutant KJΔA1ΔA2ΔB was obtained. It exhibited minimal growth in Luria–Bertani (LB) broth and could only be subcultured twice on LB agar. Therefore, the triple mutant was not included in the following study. Except for mutant KJΔA1ΔA2, the growth patterns of all single and double mutants tested were similar to that of wild-type KJ. Mutant KJΔA1ΔA2 exhibited a growth defect in LB broth, with a longer generation time and a lower plateau density of its stationary phase (Figure 2B).

The SOD activities of wild-type KJ and its derived *sod* mutants were assessed. Mutants KJ∆A2, KJ∆A1∆B, and KJ∆A2∆B exhibited half to two-thirds of the SOD activity compared to wild-type KJ. However, the SOD activity was moderately elevated in the case of *sodA1* inactivation (Figure 2C).

Given the possible functional redundancy of the three enzymes, we considered whether compensatory expression occurs when one or two SODs are inactivated. To test this possibility, the three *sod* mRNA transcripts in all the constructed single or double mutants were assessed by qRT–PCR. Single or combined deletion of *sodA1* and *sodB* did not affect the expression of the remaining *sod* gene(s) (Figure 2D). The most obvious compensation was observed in *sodA1* expression when *sodA2* was deleted either in KJΔA2 or in KJΔA2ΔB (Figure 2D).

### 2.3. Expression of the Three SODs in Response to Menadione and Metal Stresses

**Figure 3:**
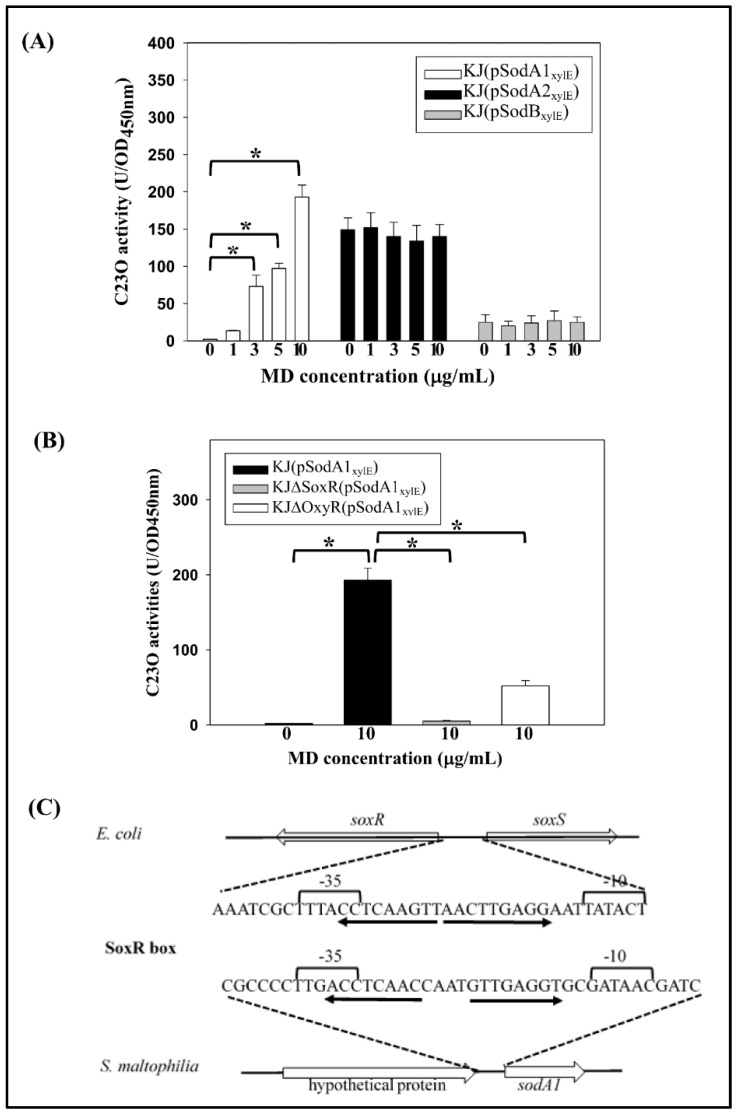
SodA1 is inducibly expressed by menadione (MD). (**A**) *Sod* gene expression under different concentrations of MD. Overnight cultured KJ(pSodA1_xylE_), KJ(pSodA2_xylE_), and KJ(pSodB_xylE_) were inoculated into fresh LB medium containing MD of different concentrations as indicated at an initial OD_450nm_ of 0.15. C23O activities were determined after a 6-h culture. Bars represent the mean from three independent experiments. Error bars represent the standard error (SD) of the mean. Data are expressed as mean ± SD (*N* = 3). * *p* < 0.01, as calculated by the Student’s *t*-test. (**B**) Regulatory roles of SoxR and OxyR in MD-mediated *sodA1* up-expression. Plasmid pSodA1_xylE_ was transported into wild-type KJ and its isogenic *oxyR* and *soxR* mutants, KJΔOxyR and KJΔSoxR, respectively. C23O activities of these recombinant strains were determined with or without 6-h MD challenge. Bars represent the mean from three independent experiments. Error bars represent the standard error (SD) of the mean. Data are expressed as mean ± SD (*N* = 3). * *p* < 0.01, as calculated by the Student’s *t*-test. (**C**) Similarity between the SoxR boxes of the *E. coli soxR* promoter and the *S. maltophilia sodA1* promoter. The palindrome in the SoxR box is indicated by solid arrows.

The expression of SODs is regulated by superoxide in several bacteria [3,8] Given that superoxide cannot freely diffuse across membranes, we used menadione (MD) as a stressor to increase the intracellular superoxide concentration. MD is a redox-cycling compound that functions as a continuous source of superoxide in the cell as a consequence of repeated cycles of oxidation and reduction. The impact of excess superoxide on the expression of the three cytosolic *sod* genes was assessed. Considering the *sod* gene expression patterns shown in Figure 2A, we assessed the expression of *sod* genes at a time point of 6 h in the following experiment. The expression of *sodA1* was directly proportional to the MD concentrations ranging from 0 to 10 µg/mL. By contrast, there was no significant increase in the expressions of *sodA2* and *sodB* upon MD challenge (Figure 3A).

The impact of iron and manganese on *sod* gene expression was assessed and the results demonstrated that the expression of the three *sod* genes were unaffected by iron or manganese challenges (data not shown).

In gram-negative bacteria, SoxR and OxyR are globular regulators involved in the regulation of several oxidative stress alleviation-associated genes [14]. Therefore, the effect of SoxR and OxyR in MD-mediated *sodA1* expression was assessed. Plasmid pSodA1_xylE_ was transported into the *soxR* mutant, KJ∆SoxR, and *oxyR* mutant, KJ∆OxyR, respectively. The C23O activities expressed by these recombinant strains were investigated. The C23O activity of KJ(pSodA1_xylE_) was obviously increased upon the MD challenge and this increment was totally abolished or partially compromised when the *soxR* or *oxyR* was inactivated, respectively (Figure 3B). Thus, SoxR was essential for MD-mediated *sodA1* up-regulation, and OxyR further facilitated this process.

The SoxR binding site sequence (SoxR box) and its location in relation to the target promoter motifs are remarkably well conserved in diverse groups of gram-negative bacteria [15]. In view of the role of SoxR in MD-mediated *sodA1* induction, the presence of the SoxR box in the vicinity of the *sodA1* promoter was examined. The inverted repeat sequence 5′ACCTCAACCAATGTTGAGGT3’ was found in the vicinity of the *sodA1* promoter (Figure 3C), and this observation supports the hypothesis that oxidized SoxR directly up-regulates *sodA1* expression by binding to the SoxR box. However, no possible SoxR box was identified in the vicinities of the *sodA2* and *sodB* promoters.

### 2.4. Implication of the Three SODs in Low-Iron and/or Superoxide Stress Conditions

**Figure 4:**
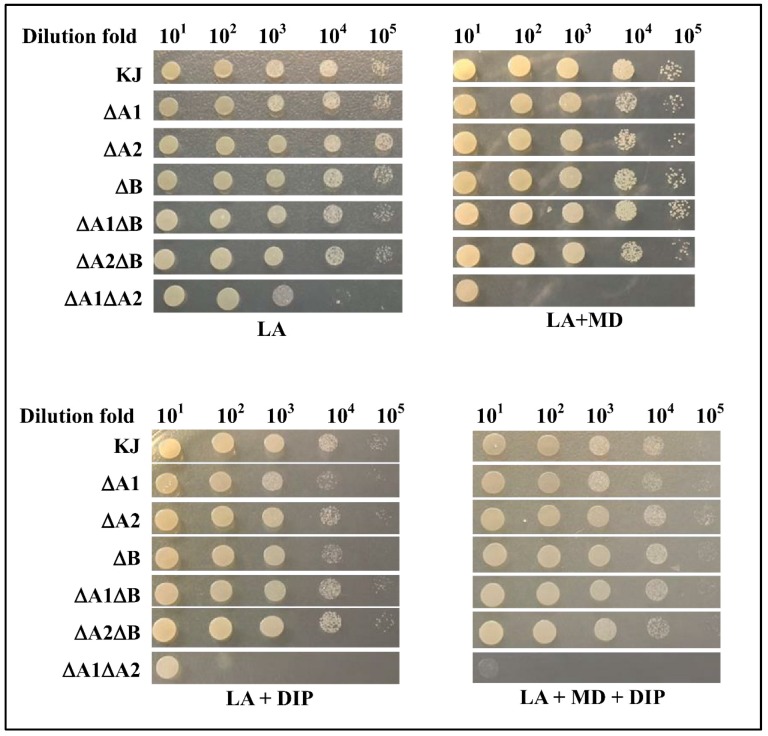
Implication of three SODs in low-iron and/or superoxide stress conditions. The logarithmic-phase bacterial cells tested were collected, adjusted to a concentration of 2 × 10^5^ CFU/µL, and 10-fold serially diluted. Then 5 µL of bacterial cells was spotted onto the LB agar (LA) containing 10 μg/mL MD and/or 30 μg/mL iron chelator 2,2-dipyridyl (DIP). After 24 h of incubation at 37 °C, the growth of bacterial cells was observed.

Innate immunity and nutritional immunity are two main defense mechanisms for mammalian hosts to confront bacterial infections. Nutritional immunity is achieved by sequestering available metals such as Mn, Fe, and Cu away from bacteria [16]. Given that the activities of SodA and SodB rely on Mn and Fe, respectively, it is of interest to evaluate the abilities of SODs to protect the bacteria against superoxide stress in metal depletion conditions. A low-iron condition can be achieved by adding the iron chelator 2,2-dipyridyl (DIP). The roles of SODs in superoxide excess alone, low-iron alone, or combined conditions were investigated. Except for KJ∆A1∆A2, the bacterial viability of all mutants tested was minimally affected in the MD-containing LB agars, and slightly compromised in DIP- and MD/DIP-containing LB agars compared to that of wild-type KJ (Figure 4). However, compared to that in LB agar, the cell viability of KJ∆A1∆A2 was significantly compromised in MD- or DIP-containing LB agars. Simultaneous additions of MD and DIP led to worse viability than in either condition alone (Figure 4).

### 2.5. Implication of the Three SODs in Low-Mn and/or Superoxide Stress Conditions

**Figure 5:**
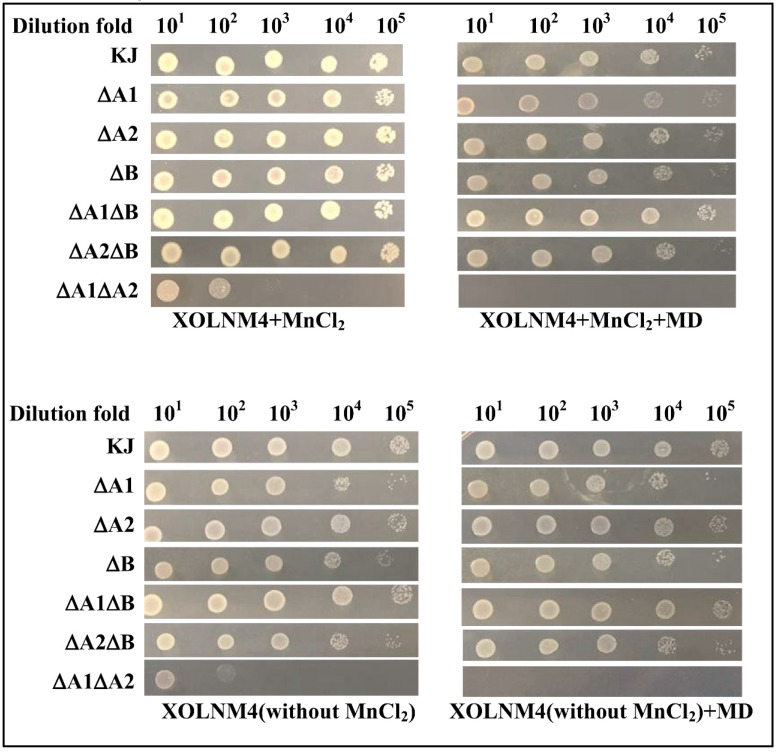
Implication of three SODs in low-manganese and/or superoxide stress conditions. The logarithmic-phase bacterial cells tested were collected, adjusted to a concentration of 2 × 10^5^ CFU/µL, and 10-fold serially diluted. Then 5 µL of bacterial cells was spotted onto the XOLNM-based minimal medium and the additives of MnCl_2_ (6 µM) and MD (10 µg/mL) as indicated. After 24 h of incubation at 37 °C, the growth of bacterial cells was observed.

Given that manganese is crucial for SodA enzyme activity, the roles of SODs in superoxide excess, low-Mn, or combined conditions were also considered. Since there is no commercially available Mn chelator, we used the XOLNM-based minimal medium with or without MnCl_2_ for investigating the impact of Mn limitation. Except for KJΔA1ΔA2, there was a slight difference in cell viability between wild-type KJ and all mutants tested (Figure 5). The cell viability of KJΔA1ΔA2 was compromised either in MD-containing medium (XOLNM4+MD) or in low-manganese medium (XOLNM4-Mn) (Figure 5).

## 3. Discussion

For aerobically grown bacteria, superoxide is an inevitable stress. To cope with the stress, a bacterium generally harbors at least two different SOD enzymes and these SOD enzymes exquisitely collaborate. It is generally known that some SODs are only sufficient to protect bacteria from aerobic metabolism-generated superoxide. When more superoxide is generated by exogenous stimuli, bacteria will trigger one or more additional SOD enzyme(s) to cope with the increased superoxide stress [17]. Nevertheless, the significances of MnSOD and FeSOD are considerably diversified among bacterial species. *E. coli* harbors two cytosolic SODs, SodA and SodB. SodB is constitutively expressed and is thought to play a major role in constant defense against oxygen toxicity and alleviation of aerobic metabolism-generated superoxide. In contrast, expression of SodA is induced by exogenous stimuli and appears to adjust the cell response to the excess radical oxygen challenge [18]. SodA in *E. coli* is in part considered a rescue isoenzyme to compensate for a shortage of SodB. In *P. aeruginosa*, SodB is constitutively expressed and more important than SodA for aerobic logarithmic-phase growth [19]. However, SodA is absent in the logarithmic phase of aerobic cultures, but is specifically expressed in the stationary phase [20]. Unlike that of *E. coli*, SodA of *P. aeruginosa* does not appear to be a rescue enzyme, and has the specific role of dealing with the oxidative stress of the stationary phase. In this study, we found that *S. maltophilia* utilizes three SODs (SodA1, SodA2, and SodB) to limit the damages caused by aerobic metabolism- and exogenous stimuli-generated superoxide. SodA2 and SodB are responsible for mitigation of aerobic metabolism-generated superoxide, and SodA2 is the dominant contributor. Even in the absence of SodB, the activity of SodA2 is sufficient to handle aerobic metabolism-generated superoxide (Figure 2B). Simultaneous inactivation of *sodA1* and *sodA2* significantly affected bacterial aerobic growth (Figure 2B), indicating that SodB alone is not potent enough to manage aerobic metabolism-generated superoxide. Collectively, our results support that SodA2, rather than SodA1 or SodB, is the key contributor for the alleviation of aerobic metabolism-mediated superoxide, similar to SodB proteins in *E. coli* [18] and *P. aeruginosa* [19]. SodA1, like the SodA of *E. coli* [18], appears to be a rescue enzyme. Although SodA1 is not obviously expressed in aerobic-growth bacteria, it can be compensatively expressed when SodA2 activity is lost (Figure 2D).

A more interesting observation revealed in this study was that SodB of *S. maltophilia* appears to be marginalized with respect to superoxide stress alleviation. In all assayed conditions in this study, including physiologically aerobic growth, MD stress challenge, and manganese- or iron-depleted conditions, loss of SodB function barely affects bacterial viability. Recent studies have pointed out that SODs may possess new functions beyond their conventional role in superoxide radical scavenging, including acting as transcriptional factors, RNA binding proteins, and signal modulators [21]. Therefore, SodB may have some unidentified functions beyond eliminating superoxide radicals. Given the abatement of SodB function in superoxide dismutation, SodA1 may be an evolutionary substitute to assist SodA2 to cope with excess superoxide stress.

**Figure 6:**
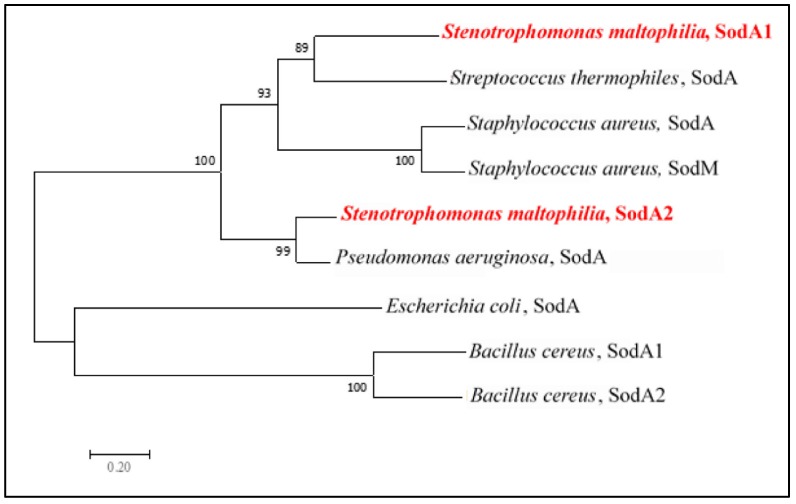
Phylogenetic relationship of MnSODs (SodA1 and SodA2) of *S. maltophilia* and previously characterized MnSODs in other bacteria. The dendrogram was inferred by the neighbor-joining method using the amino acid sequences of the proteins. The bootstrap numbers at the branch points refer to 1000 replications.

MnSOD is encoded by a single gene in the majority of bacteria [18,20]. Similar to *S. maltophilia* which harbors two genes encoding two MnSODs, the only other reported cases with two *Mnsod* genes are in *Bacillus* spp. [10,11] and *S. aureus* [8]. The phylogenic relationship of MnSODs among different species was analyzed; relative to other MnSODs assayed, the two MnSODs in *B. cereus* or *S. aureus* were phylogenetically closed (Figure 6) and conversely, the two MnSODs of *S. maltophilia* were located in distinct phylogenetic clades. SodA1 and SodA2 of *S. maltophilia* were phylogenetically close to the SodAs of *S. thermophiles* and *P. aeruginosa*, respectively (Figure 6), implying that the two *sodA* genes of *S. maltophilia* may have evolved by gene capture from different bacteria, rather than by duplication of the original gene. *S. maltophilia* is the first reported gram-negative bacterium with two *sodA* genes. We further examined the conservation of the two-SodA system in the available sequenced *S. maltophilia* genomes, and found that the two-SodA system is well conserved in the sequenced *S. maltophilia* assayed.

*S. maltophilia* is a common isolate from cystic fibrosis (CF) patients [12]. In the lungs of CF patients, the bacteria are exposed to ROS produced by hosts’ macrophages. In addition, ionic dyshomeostasis has also been observed in CF patients, with reduced levels of Mn and Fe [22]. Therefore, synergism between iron/manganese sequestration and oxidative burst is a critical challenge for *S. maltophilia* infection. Based on the findings in this study, MnSODs (SodA1 and SodA2) are likely to better enable bacterium to cope with the oxidative burst or metal sequestration than FeSOD (SodB). SodA1 or SodA2 alone exhibit competent ability to cope with stresses; nevertheless, SodB alone is not potent enough. When moderate stress is encountered, the constitutively expressed SodA2 can respond sufficiently. SodA2 is sustained by the inducibly expressed SodA1 when a high level of stress occurs. The presence of two MnSODs provides *S. maltophilia* the flexibility to deal with superoxide stresses of different levels and the ability to survive when one of the MnSODs is defective.

## 4. Materials and Methods

### 4.1. Bacterial Strains, Plasmid, and Growth Condition

Table S1 lists the bacterial strains, plasmids, and PCR primers used in this study. *S. maltophilia* and *E. coli* were grown aerobically in Luria–Bertani (LB) broth or on LB agar at 37 °C. XOLN minimal medium contained the basal salts (per litre): K_2_HPO_4_, 0.7 g; KH_2_PO_4_, 0.2 g; (NH_4_)_2_SO_4_, 1.0 g; yeast extract, 0.625 g; tryptone, 0.625 g, MgCl_2_6H_2_O, 0.1 g; FeSO_4_7H_2_O, 0.01 g, pH 7.15. XOLNM was formulated XOLN [23] minimal medium with 4% maltose as a carbon source.

### 4.2. Construction of Deletion Mutants KJΔA1, KJΔA2, and KJΔB

The *sodA1*, *sodA2*, and *sodB* genes were deleted from the chromosome of *S. maltophilia* KJ using the two-step allelic exchange strategy. Plasmids pΔSodA1, pΔSodA2, and pΔSodB were prepared for the construction of KJΔA1, KJΔA2, and KJΔB respectively. Two PCR amplicons, corresponding to upstream and downstream of the gene intended to be deleted, were amplified using the paired primer sets (SodA1N-F/SodA1N-R and SodA1C-F/SodA1C-R for *sodA1*, SodA2N-F/SodA2N-R and SodA2C-F/SodA2C-R for sodA2, and SodBN-F/SodBN-R and SodBC-F/SodBC-R for *sodB*) (Table S1), and subsequently cloned into pEX18Tc to yield the recombinant plasmids pΔSodA1, pΔSodA2, and pΔSodB. Plasmids pΔSodA1, pΔSodA2, and pΔSodB were mobilized into KJ cells by conjugation, and transconjugant selection was performed as descried previously [24]. PCR analysis and DNA sequencing were performed to confirm the correctness of mutants. Double and triple mutants were constructed from single mutants by the same procedure.

### 4.3. SOD Activity Assay

Overnight-cultured bacterial cells were inoculated into fresh LB broth and further cultured for 7 h. The mid-log phase bacterial cells were collected and resuspended into 10 mM phosphate buffer (pH 7.0). After sonication, the supernatant was collected and assayed for SOD activity using the RANSOD kit (Randox Labs, Crumlin, UK) according to the manufacturer’s instructions. The method employs xanthine and xanthine oxidase (XOD) to generate superoxide radicals, which react with 2-(4-iodophenyl)-3-(4-nitrophenol)-5-phenyltetrazolium chloride (INT) to form a red formazan dye. The SOD activity is measured by the degree of inhibition of this reaction. One unit of SOD is defined as a 50% inhibition of the rate of reduction of INT under the assay condition. Protein concentration was determined by the Bradford protein assay (Bio-Rad, Hercules, CA, USA). SOD activity was expressed as unit per mg protein of cell extract (U/mg).

### 4.4. Construction of Promoter–xylE Transcriptional Fusion Plasmids, pSodA1_xylE_, pSodA2_xylE_, and pSodB_xylE_

The promoter regions of *sodA1*, *sodA2*, and *sodB* genes were obtained by PCR using the primer sets SodA1N-F/SodA1N-R, SodA2N-F/SodA2N-R, and SodBN-F/SodBN-R respectively. Transcriptional fusion reporters for *sodA1*, *sodA2*, and *sodB* were constructed by cloning the promoter region of each gene in front of the *xylE* gene in pRKXylE [25], yielding pSodA1_xylE_, pSodA2_xylE_, and pSodB_xylE_.

### 4.5. Determination of C23O Activity

The product of the *xylE* gene, catechol 2, 3-dioxygenase (C23O), catalyzes the hydrolysis of catechol into the yellow 2-hydroxymuconate semialdehyde, which can be quantitatively determined by spectrophotometric analysis at a wavelength of 375 nm. The C23O activity was determined spectrophotometrically at 375 nm after the addition of 100 mM catechol, as described previously [26]. The rate of hydrolysis was calculated by using 44,000 M^−1^cm^−1^ as the extinction coefficient. One unit of enzyme activity (U) was defined as the amount of C23O that converts 1 nanomole of catechol per min. The C23O activity was expressed as U/OD_450nm_.

### 4.6. Quantitative Real-Time PCR (qRT–PCR)

The expression levels of the genes of interest were measured by qRT–PCR. Total RNA isolation, cDNA preparation, and qRT–PCR were carried out as described previously [27]. The primers used for qRT–PCR are listed in Table S1. The relative levels of expression were calculated using the threshold cycle ΔΔ*C*t method [28]. The expression of 16S rRNA was used for normalizing the results. Three independent replicates were done in each assay.

### 4.7. Cell Viability Assay

The logarithmic-phase bacterial cells tested were collected, adjusted to a concentration of 2 × 10^5^ CFU/µL, and 10-fold serially diluted. Then 5 μL of bacterial cells was spotted onto the agar indicated. After 24 h of incubation at 37 °C, the growth of bacterial cells was observed.

### 4.8. Sequence and Phylogenetic Analyses

The NCBI BLAST tool (http://www.ncbi.nlm.nih.gov) was used to search for protein sequences of MnSODs of *E. coli*, *P. aeruginosa*, *S. aureus*, *B. cereus*, and *S. thermophile*. Multiple sequence alignments were performed using the ClustalW 2.1 program. Phylogenetic analysis was conducted using the neighbor-joining method. The dendrogram was inferred by the neighbor-joining method using the amino acid sequences of the proteins. The bootstrap numbers at the branch points refer to 1000 replications. The tree is drawn to scale, with branch lengths in the same units as those of the evolutionary distances used to infer the phylogenetic tree. The evolutionary distances were computed using the Poisson correction method and are in the units of the number of amino acid substitutions per site.

## Supplementary Materials

Supplementary materials can be found at https://www.mdpi.com/1422-0067/20/7/1770/s1.
