# Supplementary material for: Roles of the Two-MnSOD System of Stenotrophomonas maltophilia in the Alleviation of Superoxide Stress
Source: Int J Mol Sci. 2019 Apr 10;20(7):1770. doi: 10.3390/ijms20071770 (PMC6479884; doi:10.3390/ijms20071770)
Supplement: Supplementary file 1 [file ijms-20-01770-s001.pdf]

Table 1. Bacterial strains, plasmids and primers used in this study.

Strain, plasmid, or primer	Genotype or properties	Reference
S. maltophilia		
KJ	Wild type, a clinical isolate from Taiwan	1
KJ A1	*S. maltophilia* KJ *sodA1* mutant; *sodA1*	This study
KJ A2	*S. maltophilia* KJ *sodA2* mutant; *sodA2*	This study
KJ B	*S. maltophilia* KJ *sodB* mutant; *sodB*	This study
KJ A1ΔB	*S. maltophilia* KJ *sodA1* and *sodB* double mutant; *sodA1, sodB*	This study
KJ A2ΔB	*S. maltophilia* KJ *sodA2* and *sodB* double mutant; *sodA2, sodB*	This study
KJ A1 A2	*S. maltophilia* KJ *sodA1* and *sodA2* double mutant; *sodA1, sodA2*	This study
KJ A1ΔA2ΔB	*S. maltophilia* KJ *sodA1*, *sodA2*, and *sodB* triple mutant; *sodA1, sodA2, sodB*	This study
KJΔSoxR	*S. maltophilia* KJ *soxR* mutant; *soxR*	2
KJΔOxyR	*S. maltophilia* KJ *oxyR* mutant; *oxyR*	2
Escherichia coli		
DH5α	F- ϕ80d*lacZΔM15* Δ(*lacZYA-argF*)*U169 deoR recA1 endA1 hsdR17* (r_{k}^{-} m_{k}^{+}) *phoA supE44λ⁻ thi-1 gyrA96 relA1*	Invitrogen
S17-1	λ pir + mating strain	3
**Plasmids**		
pEX18Tc	*sacB oriT*, Tc^{r}	4
pRK415	Broad host range expression vector, Tc^{r}	5
pRKXylE	Plasmid pRK415 containing a *xylE* cassette and the orientation of *xylE* gene being opposite to the orientation of *lacZ* promoter of pRK415; Tc^{r}	1
p SodA1	pEX18Tc with an internal deletion *sodA1* gene; Tc^{r}	This study
pΔSodA2	pEX18Tc with an internal deletion *sodA2* gene; Tc^{r}	This study
pΔSodB	pEX18Tc with an internal deletion *sodB* gene; Tc^{r}	This study
$pSodA1_{xylE}$	pRK415 with a P_{sodA1}-*xylE* promoter transcriptional fusion; Tc^{r}	This study
$pSodA2_{xylE}$	pRK415 with a P_{sodA2}-*xylE* promoter transcriptional fusion; Tc^{r}	This study
$pSodB_{xylE}$	pRK415 with a P_{sodB}-*xylE* promoter transcriptional fusion; Tc^{r}	This study
**Primers**		
SodA1N-F	5′- GGGAGCTCAGTTGAAGGCGTGGA -3′	This study
SodA1N-R	5′- GCTCTAGAAGCGAGTACGACAT -3′	This study
SodA1C-F	5′- GATTCTAGAACTGGATGTCTGGGA -3′	This study
SodA1C-R	5′- CGCGCATGCGGATCGGCAAA -3′	This study
SodA2N-F	5′- GCGGTACCTGCTGTTCGCGGTTA -3′	This study
SodA2N-R	5′- CATCTAGAGCAGGGTATAGGCCA -3′	This study
SodA2C-F	5′- CGTTCTAGAACGTCATCGACTGGA -3′	This study
SodA2C-R	5′- ACCGTCGACCTGATGACCTGGAT -3′	This study
SodBN-F	5′-GGGAGCTCGGCAGGTTGGAGA-3′	This study
SodBN-R	5′-GAAAGCTTCCACGTAGGCGCGA-3′	This study
SodBC-F	5′-GGGAGCTCGGCAGGTTGGAGA-3′	This study

SodBC-R	5′-CAGGTCGACCACCTCGATGCGA-3′	This study
SodA1Q-F	5′- TACTCGCTTCCCCCGCTCCC -3′	This study
SodA1Q-R	5′- GCTGAGCACGGTCCAGAACAGG -3′	This study
SodA2Q-F	5′- CCTACGCCTACGACGCGCTG -3′	This study
SodA2Q-R	5′- CACGTCGCCCACCGGGTTAC -3′	This study
SodBQ-F	5′- TGCCTGCCCTGCCCTACCTTTC -3′	This study
SodBQ-R	5′- TCGCCGAACTGGCGCTTCAC -3′	This study
16S DNA-F	5′- GACCTTGCGCGATTGAATG -3′	6
16S DNA-R	5′- CGGATCGTCGCCTTGGT -3′	6

References

1. Hu, R.M.; Huang, K.J.; Wu, L.T.; Hsiao, Y.J.; Yang, T.C. Induction of L1 and L2 β-lactamases of *Stenotrophomonas maltophilia*. *Antimicrob. Agents Chemother.* **2008**, 52, 1198–1200.
2. Wu, C.J.; Chiu, T.T.; Lin, Y.T.; Huang, Y.W.; Yang, T.C. Role of *smeU1VWU2X* operon in the alleviation of oxidative stresses and the occurrence of sulfamethoxazole/trimethoprim-resistant mutants in *Stenotrophomonas maltophilia*. *Antimicrob. Agents Chemother.* **2018**, 62, e02114-17.
3. Simon, R.; O'Connell, M.; Labes, M.; Puhler, A. Plasmid vector for the genetic analysis and manipulation of *Rhizobia* and other Gram-negative bacteria. *Methods Enzymol* **1986.** 118, 640-659.
4. Hoang, T.T.; Karkhoff-Schweizer, R.R.; Kutchma, A.J.; Schweizer, H.P. A broad-host-range Flp-FRT recombination system for site-specific excision of chromosomally-located DNA sequences: application for isolation of unmarked *Pseudomonas aeruginosa* mutants. *Gene* **1998**, 212**,** 77-86.
5. Keen, N.T.; Tamaki, S.; Kobayashi, D.; Trollinger, D. Improved broad-host-range plasmids for DNA cloning in gram-negative bacteria. Gene **1988,** 70, 191-197.
6. Chen, C.H.; Huang, C.C.; Chung, T.C.; Hu, R.M.; Huang, Y.W.; Yang, T.C. Contribution of resistance-nodulation-division efflux pump operon *smeU1-V-W-U2-X* to multidrug resistance of *Stenotrophomonas maltophilia*. *Antimicrob. Agents Chemother.* **2011**, 55, 5826-5833.
